# Endogenous and environmental signals in regulating vascular development and secondary growth

**DOI:** 10.3389/fpls.2024.1369241

**Published:** 2024-04-02

**Authors:** Huanzhong Wang

**Affiliations:** ^1^ Department of Plant Science & Landscape Architecture, University of Connecticut, Storrs, CT, United States; ^2^ Institute for System Genomics, University of Connecticut, Storrs, CT, United States

**Keywords:** secondary growth, cambium, peptide signal, hormones, environmental factors

## Introduction

**Figure 1:**
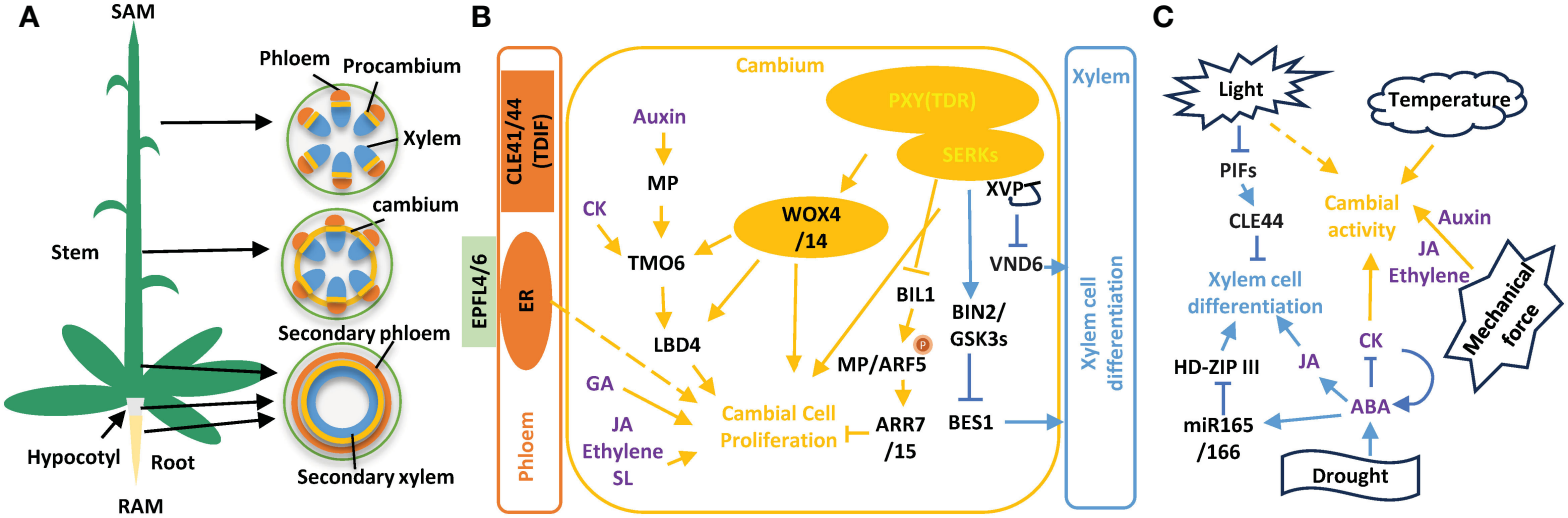
Vascular development and cambial activity is regulated by endogenous programs and exogenous signals. **(A)** Vascular development in *Arabidopsis* stem, hypocotyl, and root organs. Young stem develops discrete vascular bundles comprised of phloem, xylem, and intervening pro-cambium. Developing stems form cambial cells at vascular and interfascicular regions. Secondary growth produce secondary phloem and secondary xylem in mature stem, hypocotyl, and root. **(B)** Short-range peptide signals, TDIF-PXY-WOX4 and EPFL4/6-ERECTA modules, and hormonal signaling pathways regulate cambium cell proliferation and xylem cell differentiation. **(C)** Environmental factors regulate vascular development.

Resilient plant growth depends on the function of meristems, including the shoot apical meristem (SAM), the root apical meristem (RAM), and lateral meristems. The vascular cambium is a lateral meristem responsible for secondary growth and stem expansion at the radial axis. The vascular cambium harbors stem cells that proliferate, and progenies differentiate into xylem and phloem cells. Each radial cell file has one bifacial stem cell that produces both xylem and phloem cell lineages (Shi et al., 2019; Smetana et al., 2019). Cambial stem cells and undifferentiated xylem and phloem progenitors form a cambial region, which is often used as an indicator of cambial activity (**Figure 1A**). The apical meristems and vascular meristems are spatially separated. Coordinated growth between these meristems is mediated through mobile signals, such as hormones, peptides, and mechanical cues (Fischer et al., 2019). Environmental factors also played important roles in tuning the secondary growth.

Secondary growth is an evolutionary innovation, providing sufficient mechanical support and efficient long-distance fluid transport for larger and more complex plant bodies (Tonn and Greb, 2017). Additionally, secondary growth produces large amounts of woody biomass, recalcitrant forms of carbon that can potentially mitigate global warming by fixing atmospheric carbon into storage. The primary vascular development is established early during embryogenesis (Miyashima et al., 2013). Pre-procambial initials start dividing at the globe stage, forming a radial pattern resembling post-embryonic root vasculature (Rodriguez-Villalon et al., 2014). The signaling pathways regulating primary vascular development were discussed in several recent excellent review papers (Fischer and Teichmann, 2017; Tonn and Greb, 2017; Wang, 2020; Turley and Etchells, 2022; Wang et al., 2023). This paper mainly focuses on advances in regulating plant vascular cambial activity and secondary growth.

## Short-range regulatory pathways in secondary growth

### The peptide-receptor module CLE41/44-PXY plays a central role in secondary growth

The proliferation of vascular stem cells and subsequent differentiation of progeny cells are tightly regulated to ensure the proper organization of vascular tissues. Among the known regulatory pathways, the TRACHEARY ELEMENT DIFFERENTIATION INHIBITORY FACTOR (TDIF) peptide and its receptor PHLOEM INTERCALATED WITH XYLEM (PXY), also known as TDIF RECEPTOR (TDR), form the most important and best studied short-range signal in secondary growth. PXY is a member of the receptor-like kinases (RLKs) with 21 leucine-rich repeats (LRRs) and is explicitly expressed on the xylem side of the vascular cambium (Fisher and Turner, 2007; Hirakawa et al., 2008; Etchells and Turner, 2010; Shi et al., 2019). Interestingly, the TDIF ligand coding genes, *CLAVATA3/ENDOSPERM SURROUNDING REGION 41* (*CLE41*), *CLE42*, and *CLE44* are expressed in the phloem (Ito et al., 2006; Hirakawa et al., 2008; Etchells and Turner, 2010). The TDIF dodecapeptide is produced from the cleavage of much longer pre-peptides through unknown mechanisms (Ito et al., 2006) and can bind to the inner concave surface of the LRR domain of the PXY receptor (Morita et al., 2016; Zhang et al., 2016a). The function of the ligand–receptor pair of TDIF-PXY requires co-receptors SOMATIC EMBRYOGENESIS RECEPTOR KINASEs (SERKs) to activate downstream pathways (Zhang et al., 2016b). Other membrane-localized partners, such as xylem differentiation and vascular patterning (XVP), may modulate TDIF-PXY function by forming protein complexes with PXY-SERKs coreceptors (Yang et al., 2020a) (**Figure 1B**).

The TDIF signal and its downstream components regulate cambial cell proliferation, xylem cell differentiation, and vascular patterning. First, the TDIF-PXY binding activates the cambium-expressed *WUSCHEL-RELATED HOMEOBOX* (*WOX*) transcription factor genes, *WOX4* and *WOX14*, and enhances cambial cell proliferation (Hirakawa et al., 2010; Etchells et al., 2013). In contrast, the mutation of *WOX4* and *WOX14* reduces the number of cells in root and stem vascular bundles (Etchells et al., 2013; Zhang et al., 2019). Additionally, the TDIF-PXY module inhibits BIN2 LIKE 1 (BIL1) activity, which phosphorylates MONOPTEROS (MP)/AUXIN RESPONSE FACTOR 5(ARF5) and upregulates negative regulators of cytokinin signaling ARABIDOPSIS RESPONSE REGULATOR 7 (ARR7) and ARR15 (Han et al., 2018), connecting auxin-cytokinin signaling to maintain cambial activity. Second, the TDIF-PXY module represses xylem cell differentiation, as shown by ectopic xylem differentiation and lignification of parenchyma cells in the *pxy* mutant (Etchells et al., 2016). Brassinosteroid (BR) signaling likely mediates TDIF-PXY signal in repressing xylem cell differentiation, as shown by PXY interaction with BRASSINOSTEROID INSENSITIVE 2 (BIN2), which phosphorylates and promotes the degradation of BRASSINAZOLE RESISTANT 1(BZR1) and BRI1-EMS-SUPPRESSOR 1 (BES1) (Kondo et al., 2014; Saito et al., 2018). Lastly, TDIF-PXY signal controls vascular patterning, the organization of phloem, procambium, and xylem cells (Fisher and Turner, 2007; Etchells and Turner, 2010). Until recently, the LATERAL ORGAN BOUNDARIES DOMAIN 4 (LBD4) was indicated as the TDIF-PXY downstream component in regulating vascular patterning (Zhang et al., 2019; Smit et al., 2020). LBD4 is part of a feedforward loop downstream of PXY, mediating cell proliferation and vascular bundle shape, i.e., tangential:radial axis ratio, in inflorescence stems (Smit et al., 2020; Turley and Etchells, 2022) (**Figure 1B**). It appears that the functions of TDIF-PXY signaling are conserved because homologs of the *Arabidopsis CLE41* and *WOX4* genes play similar functions in *Populus* (Kucukoglu et al., 2017, Kucukoglu et al., 2020).

### ERECTA and other receptor-like kinases participate in secondary growth

In addition to PXY and its homologous PXL1 and PXL2 (Fisher and Turner, 2007; Etchells et al., 2013), several other LRR-RLKs have been identified as regulators in vascular development (Agusti et al., 2011b; Uchida and Tasaka, 2013; Wang et al., 2013; Gursanscky et al., 2016). Among these LRR-RLKs, ERECTA (ER) and its two homologous proteins, ERL1 and ERL2 (Shpak et al., 2004), regulate vascular development and fiber formation in the stem (Ragni et al., 2011; Etchells et al., 2012; Uchida and Tasaka, 2013). Mutation of all three ER family (ERf) genes resulted in fewer cells in stem vascular bundles (Etchells et al., 2013). Furthermore, phloem-specific expression of *ER* can complement the defects in the procambium of the *er erl1* mutant plants (Uchida and Tasaka, 2013). In the same study, the ligands for ERf proteins, EPIDERMAL PATTERNING FACTOR LIKE 4 (EPFL4) and EPFL6, were found to be expressed in the endodermis (Uchida et al., 2012; Uchida and Tasaka, 2013). The downstream components of the ligand-receptor of the EPFL4/6-ERf pair have yet to be identified (**Figure 1B**).

There are indications that TDIF-PXY signaling interacts with EPFL-ERf signaling in vascular development. The *pxy er* double mutant has fewer cells in vascular bundles and shows a much stronger phenotype than either *pxy* or *er* mutant, indicating genetic interaction between PXY and ER signaling (Etchells et al., 2012; Wang et al., 2019). Indeed, expression analyses showed cross-regulation between these two pathways (Wang et al., 2019). The mechanism of the cross-regulation between PXY and ER is elusive, although there is known protein–protein interaction between PXY and ERf (Smakowska-Luzan et al., 2018; Mott et al., 2019) and convergence of downstream common genes, such as the *WOX4* gene (Wang, 2020; Turley and Etchells, 2022).

## Developmental programs and hormonal signals in secondary growth

### Cytokinin, auxin, and gibberellin regulate secondary growth

Cambial activity is influenced by signals from the apical meristems and developmental cues through phytohormones. Removing the SAM, the main auxin source, halts secondary growth (Sundberg and Uggla, 1998), while exogenous auxins application restores cambial cell division (Agusti et al., 2011a), demonstrating the connection between apical meristems and cambial activity.

Cytokinin is critical for cambial activity as shown by the lack of cambium formation in the quadruple mutant *atipt1;3;5;7*, disrupting four ATP/ADP isopentenyltransferase (*IPT*) genes, while the application of exogenous cytokinin restored vascular cambium (Matsumoto-Kitano et al., 2008). Cytokinins initiate cambial initiation in the *Arabidopsis* root through *LBD3* and *LBD4*; at the same time, *LBD1* and *LBD11* participate in prolonged secondary growth (Ye et al., 2021). In *Populus* stems, cytokinin concentration peaks in the developing phloem cells, and overexpressing the *IPT7* gene enhances cambial activity (Immanen et al., 2016). Decreasing cytokinin levels by expressing *CYTOKININ OXIDASE/DEHYDROGENASE* 2 (*CKX2*) gene in phloem non-cell autonomously restricts cambial activity (Fu et al., 2021).

Auxin also has a crucial role in secondary growth. In *Arabidopsis* root, secondary growth starts from the divisions of the xylem-adjacent procambial cells, which function as the stem cell organizer (Smetana et al., 2019). A local maximum of the auxin and consequent expression of HD-ZIP III transcription factors promotes cellular quiescence of the organizer cells (Smetana et al., 2019). In the stem, the inhibition of polar auxin transport results in auxin accumulation at the base of stems, therefore promoting secondary growth (Suer et al., 2011). In tree stems, auxin distributes in a radial concentration gradient, with the highest concentration at the cambium zone (Uggla et al., 1996; Tuominen et al., 1997). Disruption of auxin signaling or reducing auxin responsiveness led to reduced cambial cell division (Tuominen et al., 1997; Nilsson et al., 2008).

Gibberellins (Gas) also regulate cambial activity. Either directly applying active Gas or overexpression of a gibberellin biosynthesis gene *Gibberellin 20-oxidase* (*GA20ox*) enhances cambial activity (Wang et al., 1995; Eriksson et al., 2000). Shoot-produced GAs are required for secondary growth in *Arabidopsis* hypocotyls (Ragni et al., 2011). In addition, mutants with defects in GA biosynthesis show reduced cambium activity, confirming GAs as positive regulators of secondary growth (Ragni et al., 2011).

### Ethylene, jasmonic acid, and strigolactones in secondary growth

Hormones induced by environmental fluctuation, including ethylene, jasmonic acid (JA), and strigolactone (SL), play roles in secondary growth. In *Arabidopsis*, the ethylene overproducer1 (*eto1*) plants show increased vascular size in hypocotyls and inflorescence stem (Etchells et al., 2012). A large number of ETHYLENE RESPONSE FACTOR (ERF) transcription factors, especially ERF018 and ERF109, are involved in vascular cell division. Cambial activity is enhanced in another ethylene-overproducing mutant, *acs7-d*, whose phenotype depends on *WOX4* function, indicating that TDIF and ethylene signaling converge at the WOX4 level (Yang et al., 2020b). The function of ethylene is conserved in tree species, as shown by ethylene or aminocyclopropane-1-carboxylate (ACC) treatment that promotes cambial division and wood formation (Love et al., 2009). Genome-wide transcriptional profiling indicated that components of the JA signaling pathway are positive cambium regulators in *Arabidopsis* stem (Sehr et al., 2010). Furthermore, SL stimulates cambial activity as mutations in SL signaling or biosynthesis inhibit cambial activity (Agusti et al., 2011a). It is worth noting that all these known hormonal signals positively regulate secondary growth.

## Environmental signals regulate secondary growth

### Light

Light is one of the most critical environmental signals that control various developmental processes (Jiao et al., 2007; De Wit et al., 2016). The vascular system is an evolutionary innovation for plant adaption to light competition, which theory is supported by fossil records (Beck, 1971; Stewart et al., 1993; Meyer-Berthaud et al., 2010) and computer simulation studies (Knoll and Niklas, 1987; Fitch et al., 1994). Despite the importance of light in vascular plant evolution, how light influences vascular development is not well understood. In shade conditions, plants manifested various developmental responses, including elongation of stems and petioles, and increased apical dominance (Ballaré et al., 1990). In *Arabidopsis* hypocotyls, shade increases the number and types of water-conducting tracheary elements in the vascular cylinder, which may need the function of WOX4 (Botterweg-Paredes et al., 2020). Ghosh et al. reported that blue light inactivates the expression of *Phytochrome-Interacting Factors* (*PIFs*) and *CLE44*, therefore de-repressing vascular cell differentiation (Ghosh et al., 2022). It is unclear whether procambium activity is affected by blue light (Ghosh et al., 2022). Further studies indicated that shaded light conditions with a low ratio of red to far-red light inhibit secondary cell wall thickening through a PHYB-PIF4-MYC2/MYC4 module in fiber cells of the *Arabidopsis* stem (Luo et al., 2022). Therefore, light positively affects xylary cell differentiation and secondary wall development.

### Temperature

Temperature is another environmental factor affecting many developmental processes, especially cambium reactivation and xylem differentiation in trees. Trees from temperate zones undergo seasonal vascular cambial cycles of activity and dormancy. In late winter to early spring, new cells are formed in the cambial, called cambial reactivation, which is mainly affected by temperature (Begum et al., 2013; Agustí and Blázquez, 2020). Under natural conditions, cambium reactivation in different species requires varied threshold temperatures and an accumulated number of degrees more than the threshold value, also called the cambial reactivation index (CRI) (Begum et al., 2013). Xylem differentiation often starts within 3 or 4 weeks after cambium reactivation (Rossi et al., 2007). Warm springs induce early resumption of cambial cell proliferation and an early onset of xylem differentiation (Rossi et al., 2007; Begum et al., 2008). Extensive modulation of cambial transcriptome and proteome occurs during the activity–dormancy cycle in aspen (Druart et al., 2007). Localized heating of stems during dormancy induces reactivation of the cambium in various trees, including evergreen conifers (Barnett, 1992; Oribe & Kubo, 1997; Oribe et al., 2001; Gričar et al., 2006) and poplar trees (Begum et al., 2007). These studies have established a clear relationship between temperature and morphological changes in trees, but the molecular mechanism is still lacking due to the scarcity of genetic and genomic studies.

In *Arabidopsis* leaves, high temperatures increase vein density and tracheary element number, likely facilitating higher rates of transpiration (Stewart et al., 2016). Interestingly, the Swedish ecotype exhibited more pronounced responses than the Italian ecotype, indicating that genetic variation may affect temperature response (Stewart et al., 2016). In another study, the expression of *AtPXL1*, a paralog of PXY, is induced by both cold and heat stress (Stewart et al., 2016). In addition, the *atpxl1* mutant plants showed a temperature-hypersensitive phenotype (Stewart et al., 2016). It would be interesting to study if PXY activity is essential for acclimation under fluctuating temperatures.

### Mechanical force

Plants are consistently experiencing mechanical forces, including endogenous compression resulting from growing body weight, increasing number and volume of surrounding cells, and environmental forces from wind, touch, and leaning. Among the mechanical forces, body weight has been well studied on secondary growth in the model plant *Arabidopsis* (Ko et al., 2004; Sehr et al., 2010). Using artificial weight treatment, Ko et al. found that weight induces cambial differentiation, and the weight signal relies on auxin signaling components (Ko et al., 2004). The weight-load-sensing system regulates cell-wall-related genes through transcriptional regulation in the xylem (Koizumi et al., 2009). In addition to auxin signaling, other hormonal signals, such as ABA, ethylene, and JA signaling, are also involved in body-weight-induced secondary growth in *Arabidopsis* (Sehr et al., 2010; Etchells et al., 2012; Campbell et al., 2018) (**Figure 1C**). Furthermore, ethylene controls cambial proliferation during tension wood development in *Populus* (Love et al., 2009). Therefore, mechanical cues may regulate cambial cell proliferation and subsequent cell differentiation through both auxin-dependent and auxin-independent pathways.

### Water availability

Water availability is another factor that affects secondary growth. Drought induces the biosynthesis of ABA, which regulates the differentiation and patterning of primary and secondary xylem (Ramachandran et al., 2018). In *Arabidopsis* roots, ABA treatment induced extra xylem strands. At the same time, mutants in the last steps of ABA biosynthesis, *abi2-1* and *abi3-1*, displayed discontinuous or absent xylem strands, indicating the importance of ABA in xylary wall formation (Ramachandran et al., 2018). Additionally, endodermis localized ABA non-cell autonomously regulates the xylem cell types (Ramachandran et al., 2018). It was proposed that ABA induces the biosynthesis of *miRNA165/166* in the endodermis, and then, *miRNA165/166* moves to the developing xylem cells, where the miRNAs control certain HD-ZIP III factors in regulating protoxylem and metaxylem identity (Carlsbecker et al., 2010; Ramachandran et al., 2018) (**Figure 1C**). Furthermore, ABA regulates xylem patterning and maturation via *miR165a/166b*-regulated expression of HD-ZIPIII mRNAs and associated VND7 levels in tomatoes (Bloch et al., 2019).

Drought-induced ABA signal may reduce secondary growth through interactions with other hormonal pathways. For instance, water stress and ABA treatments decrease biologically active CK contents, demonstrating a mechanism for survival under abiotic stress conditions (Bloch et al., 2019). Additionally, decreased levels of CK increased ABA sensitivity, suggesting a complex crosstalk between these two hormones (Nishiyama et al., 2011; Bloch et al., 2019). Furthermore, JA induces xylem differentiation by reducing CK-dependent promotion of cell division in the vasculature in the root (Jang et al., 2017). JA is known for its function in secondary growth in the stem (Sehr et al., 2010) and is essential to ABA accumulation in roots under water deficiency (de Ollas et al., 2015). Therefore, JA and CKs are in a signaling network regulating xylem differentiation under water stress conditions.

## Discussion and future perspectives

Recent research advances have enhanced our understanding of cambial activity control and secondary growth. The growing interest in developing environmentally resilient crops requires new knowledge of how exogenous factors influence secondary growth, especially under unfavorable conditions. Research on secondary growth faces numerous technological challenges, including difficulties in direct observation of vascular tissues, lacking genetic materials in non-model plant species, and mechanism differences in different organs (Wang, 2020; Turley and Etchells, 2022).

New technologies, such as advanced microscopy and cell-based computational modeling, will be essential to visualize and analyze cambium activity. For example, whole-mount imaging coupled with gene expression at three-dimensional (3D) domains enabled analysis at single-cell precision (Truernit et al., 2008). Tools that combine the quantitative 3D image analysis and clonal analysis may be essential to understand cambium development (Bencivenga et al., 2016). In addition, integrating cell-based computational model and the function of central cambium regulators help to determine the framework for instructing tissue organization (Lebovka et al., 2023). Lastly, pulse labeling, lineage tracing, and molecular genetic techniques have advanced our understanding on the bifacial nature of vascular stem cells in both hypocotyl and root tissues in model plants *Arabidopsis* (Shi et al., 2019; Smetana et al., 2019). The combination of these techniques will help further elucidate the mechanisms of vascular development.

Research on environmental factors in secondary growth is limited to primarily morphological observations in tree species. In the future, research should focus on investigating the perception and signaling of these environmental factors using model plants and advanced omics technologies. Dissecting the functional mechanisms of the exogenous factors on vascular development may provide new insights into the regulation of cambial activity and generate new knowledge for developing new strategies in biomass deposition and carbon reduction in the era of climate change and global warming.

## Author contributions

HW: Funding acquisition, Visualization, Writing – original draft, Writing – review & editing.

## References

[B1] Agustí J. Blázquez M. A. (2020). Plant vascular development: mechanisms and environmental regulation. Cell. Mol. Life Sci. 77, 3711–3728. doi: 10.1007/s00018-020-03496-w 32193607 PMC11105054

[B2] Agusti J. Herold S. Schwarz M. Sanchez P. Ljung K. Dun E. A.. (2011a). Strigolactone signaling is required for auxin-dependent stimulation of secondary growth in plants. Proc. Natl. Acad. Sci. U. S. A. 108, 20242–20247. doi: 10.1073/pnas.1111902108 22123958 PMC3250165

[B3] Agusti J. Lichtenberger R. Schwarz M. Nehlin L. Greb T. (2011b). Characterization of transcriptome remodeling during cambium formation identifies MOL1 and RUL1 as opposing regulators of secondary growth. PLoS Genet. 7, e1001312. doi: 10.1371/journal.pgen.1001312 21379334 PMC3040665

[B4] Ballaré C. L. Scopel A. L. Sánchez R. A. (1990). Far-Red Radiation Reflected from Adjacent Leaves: An Early Signal of Competition in Plant Canopies. Sci. (80) 247, 329–332. doi: 10.1126/SCIENCE.247.4940.329 17735851

[B5] Barnett J. R. (1992). Reactivation of the Cambium in Aesculus hippocastanum L.: A Transmission Electron Microscope Study. Ann. Bot. 70, 169–177. doi: 10.1093/OXFORDJOURNALS.AOB.A088454

[B6] Beck C. B. (1971). On the anatomy and morphology of lateral branch systems of archaeopteris. Am. J. Bot. 58, 758. doi: 10.1002/j.1537-2197.1971.tb10030.x

[B7] Begum S. Nakaba S. Bayramzadeh V. Oribe Y. Kubo T. Funada R. (2008). Temperature responses of cambial reactivation and xylem differentiation in hybrid poplar (Populus sieboldii x P. grandidentata) under natural conditions. Tree Physiol. 28, 1813–1819. doi: 10.1093/treephys/28.12.1813 19193564

[B8] Begum S. Nakaba S. Oribe Y. Kubo T. Funada R. (2007). Induction of cambial reactivation by localized heating in a deciduous hardwood hybrid poplar (Populus sieboldii x P. grandidentata). Ann. Bot. 100, 439–447. doi: 10.1093/aob/mcm130 17621596 PMC2533603

[B9] Begum S. Nakaba S. Yamagishi Y. Oribe Y. Funada R. (2013). Regulation of cambial activity in relation to environmental conditions: understanding the role of temperature in wood formation of trees. Physiol. Plant 147, 46–54. doi: 10.1111/j.1399-3054.2012.01663.x 22680337

[B10] Bencivenga S. Serrano-Mislata A. Bush M. Fox S. Sablowski R. (2016). Control of oriented tissue growth through repression of organ boundary genes promotes stem morphogenesis. Dev. Cell 39, 198–208. doi: 10.1016/j.devcel.2016.08.013 27666746 PMC5084710

[B11] Bloch D. Puli M. R. Mosquna A. Yalovsky S. (2019). Abiotic stress modulates root patterning *via* ABA-regulated microRNA expression in the endodermis initials. Development 146. doi: 10.1242/dev.177097 31399468

[B12] Botterweg-Paredes E. Blaakmeer A. Hong S. Y. Sun B. Mineri L. Kruusvee V.. (2020). Light affects tissue patterning of the hypocotyl in the shade-avoidance response. PLoS Genet. 16, e1008678. doi: 10.1371/journal.pgen.1008678 32203519 PMC7153905

[B13] Campbell L. Etchells J. P. Cooper M. Kumar M. Turner S. R. (2018). An essential role for abscisic acid in the regulation of xylem fibre differentiation. Development 145. doi: 10.1242/dev.161992 30355726

[B14] Carlsbecker A. Lee J. Y. Roberts C. J. Dettmer J. Lehesranta S. Zhou J.. (2010). Cell signalling by microRNA165/6 directs gene dose-dependent root cell fate. Nat. 465, 316–321. doi: 10.1038/nature08977 PMC2967782 20410882

[B15] de Ollas C. Arbona V. Gómez-Cadenas A. (2015). Jasmonoyl isoleucine accumulation is needed for abscisic acid build-up in roots of Arabidopsis under water stress conditions. Plant Cell Environ. 38, 2157–2170. doi: 10.1111/pce.12536 25789569

[B16] De Wit M. Galvão V. C. Fankhauser C. (2016). Light-mediated hormonal regulation of plant growth and development. Annu. Rev. Plant Biol. 67, 513–537. doi: 10.1146/annurev-arplant-043015-112252 26905653

[B17] Druart N. Johansson A. Baba K. Schrader J. Sjödin A. Bhalerao R. R.. (2007). Environmental and hormonal regulation of the activity–dormancy cycle in the cambial meristem involves stage-specific modulation of transcriptional and metabolic networks. Plant J. 50, 557–573. doi: 10.1111/j.1365-313X.2007.03077.x 17419838

[B18] Eriksson M. E. Israelsson M. Olsson O. Moritz T. (2000). Increased gibberellin biosynthesis in transgenic trees promotes growth, biomass production and xylem fiber length. Nat. Biotechnol. 187, 784–788. doi: 10.1038/77355 10888850

[B19] Etchells J. P. Provost C. M. Mishra L. Turner S. R. (2013). WOX4 and WOX14 act downstream of the PXY receptor kinase to regulate plant vascular proliferation independently of any role in vascular organisation. Development 140, 2224–2234. doi: 10.1242/dev.091314 23578929 PMC3912870

[B20] Etchells J. P. Provost C. M. Turner S. R. (2012). Plant vascular cell division is maintained by an interaction between PXY and ethylene signalling. PLoS Genet. 8, e1002997. doi: 10.1371/journal.pgen.1002997 23166504 PMC3499249

[B21] Etchells J. P. Smit M. E. Gaudinier A. Williams C. J. Brady S. M. (2016). A brief history of the TDIF-PXY signalling module: Balancing meristem identity and differentiation during vascular development. New Phytol. 209, 474–484. doi: 10.1111/nph.13642 26414535

[B22] Etchells J. P. Turner S. R. (2010). The PXY-CLE41 receptor ligand pair defines a multifunctional pathway that controls the rate and orientation of vascular cell division. Development 137, 767–774. doi: 10.1242/dev.044941 20147378

[B23] Fischer U. Kucukoglu M. Helariutta Y. Bhalzerao R. P. (2019). The Dynamics of Cambial Stem Cell Activity. Annu. Rev. Plant Biol. 70, 293–319. doi: 10.1146/ANNUREV-ARPLANT-050718-100402 30822110

[B24] Fischer U. Teichmann T. (2017). The ERECTA and ERECTA-like genes control a developmental shift during xylem formation in Arabidopsis. New Phytol. 213, 1562–1563. doi: 10.1111/nph.14440 28164341

[B25] Fisher K. Turner S. (2007). PXY, a receptor-like kinase essential for maintaining polarity during plant vascular-tissue development. Curr. Biol. 17, 1061–1066. doi: 10.1016/j.cub.2007.05.049 17570668

[B26] Fitch W. M. Ayala F. J. Niklas K. J. (1994). Morphological evolution through complex domains of fitness. Proc. Natl. Acad. Sci. 91, 6772–6779. doi: 10.1073/pnas.91.15.6772 8041696 PMC44282

[B27] Fu X. Su H. Liu S. Du X. Xu C. Luo K. (2021). Cytokinin signaling localized in phloem noncell-autonomously regulates cambial activity during secondary growth of Populus stems. New Phytol. 230, 1476–1488. doi: 10.1111/nph.17255 33540480

[B28] Ghosh S. Nelson J. F. Cobb G. M. C. Etchells J. P. De Lucas M. (2022). Light regulates xylem cell differentiation *via* PIF in Arabidopsis ll Light regulates xylem cell differentiation *via* PIF in Arabidopsis. Cell Rep. 40, 111075. doi: 10.1016/j.celrep.2022.111075 35858547 PMC9638722

[B29] Gursanscky N. R. Jouannet V. Grunwald K. Sanchez P. Laaber-Schwarz M. Greb T. (2016). MOL1 is required for cambium homeostasis in Arabidopsis. Plant J. 86, 210–220. doi: 10.1111/tpj.13169 26991973 PMC5021142

[B30] Gričar J. Zupančič M. Čufar K. Koch G. Schmitt U. Oven P. (2006). Effect of Local Heating and Cooling on Cambial Activity and Cell Differentiation in the Stem of Norway Spruce (Picea abies). Ann. Bot. 97, 943. doi: 10.1093/AOB/MCL050 16613904 PMC2803384

[B31] Han S. Cho H. Noh J. Qi J. Jung H. J. Nam H.. (2018). BIL1-mediated MP phosphorylation integrates PXY and cytokinin signalling in secondary growth. Nat. Plants 4, 605–614. doi: 10.1038/s41477-018-0180-3 29988154

[B32] Hirakawa Y. Kondo Y. Fukuda H. (2010). TDIF peptide signaling regulates vascular stem cell proliferation *via* the WOX4 homeobox gene in Arabidopsis. Plant Cell 22, 2618–2629. doi: 10.1105/tpc.110.076083 20729381 PMC2947162

[B33] Hirakawa Y. Shinohara H. Kondo Y. Inoue A. Nakanomyo I. Ogawa M.. (2008). Non-cell-autonomous control of vascular stem cell fate by a CLE peptide/receptor system. Proc. Natl. Acad. Sci. U.S.A. 105, 15208–15213. doi: 10.1073/pnas.0808444105 18812507 PMC2567516

[B34] Immanen J. Nieminen K. Smolander O. P. Kojima M. Alonso Serra J. Koskinen P.. (2016). Cytokinin and auxin display distinct but interconnected distribution and signaling profiles to stimulate cambial activity. Curr. Biol. 26, 1990–1997. doi: 10.1016/j.cub.2016.05.053 27426519

[B35] Ito Y. Nakanomyo I. Motose H. Iwamoto K. Sawa S. Dohmae N.. (2006). Dodeca-CLE as peptides as suppressors of plant stem cell differentiation. Sci. (80-.) 313, 842–845. doi: 10.1126/science.1128436 16902140

[B36] Jang G. Chang S. H. Um T. Y. Lee S. Kim J. K. Choi Y. (2017). Antagonistic interaction between jasmonic acid and cytokinin in xylem development. Sci. Rep. 7. doi: 10.1038/S41598-017-10634-1 PMC5579306 28860478

[B37] Jiao Y. Lau O. S. Deng X. W. (2007). Light-regulated transcriptional networks in higher plants. Nat. Rev. Genet. 8, 217–230. doi: 10.1038/nrg2049 17304247

[B38] Knoll A. H. Niklas K. J. (1987). Adaptation, plant evolution, and the fossil record. Rev. Palaeobot. Palynol. 50, 127–149. doi: 10.1016/0034-6667(87)90043-1 11542126

[B39] Ko J. H. Han K. H. Park S. Yang J. (2004). Plant body weight-induced secondary growth in arabidopsis and its transcription phenotype revealed by whole-transcriptome profiling. Plant Physiol. 135, 1069–1083. doi: 10.1104/pp.104.038844 15194820 PMC514141

[B40] Koizumi K. Yokoyama R. Nishitani K. (2009). Mechanical load induces upregulation of transcripts for a set of genes implicated in secondary wall formation in the supporting tissue of Arabidopsis thaliana. J. Plant Res. 122, 651–659. doi: 10.1007/s10265-009-0251-7 19582540

[B41] Kondo Y. Ito T. Nakagami H. Hirakawa Y. Saito M. Tamaki T.. (2014). Plant GSK3 proteins regulate xylem cell differentiation downstream of TDIF-TDR signalling. Nat. Commun. 5. doi: 10.1038/ncomms4504 24662460

[B42] Kucukoglu M. Chaabouni S. Zheng B. Mähönen A. P. Helariutta Y. Nilsson O. (2020). Peptide encoding Populus CLV3/ESR-RELATED 47 (PttCLE47) promotes cambial development and secondary xylem formation in hybrid aspen. New Phytol. 226, 75–85. doi: 10.1111/nph.16331 31749215 PMC7065007

[B43] Kucukoglu M. Nilsson J. Zheng B. Chaabouni S. Nilsson O. (2017). WUSCHEL-RELATED HOMEOBOX4 (WOX4)-like genes regulate cambial cell division activity and secondary growth in Populus trees. New Phytol. 215, 642–657. doi: 10.1111/nph.14631 28609015

[B44] Lebovka I. Mele B. H. Liu X. Zakieva A. Schlamp T. Gursanscky N. R.. (2023). Computational modeling of cambium activity provides a regulatory framework for simulating radial plant growth. Elife 12. doi: 10.7554/eLife.66627 PMC10069871 36897801

[B45] Love J. Björklund S. Vahala J. Hertzberg M. Kangasjärvi J. Sundberg B. (2009). Ethylene is an endogenous stimulator of cell division in the cambial meristem of Populus. Proc. Natl. Acad. Sci. U. S. A. 106, 5984–5989. doi: 10.1073/pnas.0811660106 19293381 PMC2657089

[B46] Luo F. Zhang Q. Xin H. Liu H. Yang H. Doblin M. S.. (2022). A Phytochrome B-PIF4-MYC2/MYC4 module inhibits secondary cell wall thickening in response to shaded light. Plant Commun. 3, 100416. doi: 10.1016/j.xplc.2022.100416 35927944 PMC9700123

[B47] Matsumoto-Kitano M. Kusumoto T. Tarkowski P. Kinoshita-Tsujimura K. Václavíková K. Miyawaki K.. (2008). Cytokinins are central regulators of cambial activity. Proc. Natl. Acad. Sci. U. S. A. 105, 20027–20031. doi: 10.1073/pnas.0805619105 19074290 PMC2605004

[B48] Meyer-Berthaud B. Soria A. Decombeix A. L. (2010). The land plant cover in the Devonian: A reassessment of the evolution of the tree habit. Geol. Soc Spec. Publ. 339, 59–70. doi: 10.1144/SP339.6

[B49] Miyashima S. Sebastian J. Lee J. Y. Helariutta Y. (2013). Stem cell function during plant vascular development. EMBO J. 32, 178–193. doi: 10.1038/emboj.2012.301 23169537 PMC3553377

[B50] Morita J. Kato K. Nakane T. Kondo Y. Fukuda H. Nishimasu H.. (2016). Crystal structure of the plant receptor-like kinase TDR in complex with the TDIF peptide. Nat. Commun. 7, 1–9. doi: 10.1038/ncomms12383 PMC4979064 27498761

[B51] Mott G. A. Smakowska-Luzan E. Pasha A. Parys K. Howton T. C. Neuhold J.. (2019). Map of physical interactions between extracellular domains of Arabidopsis leucine-rich repeat receptor kinases. Sci. Data 61, 1–6. doi: 10.1038/sdata.2019.25 PMC6390701 30806640

[B52] Nilsson J. Karlberg A. Antti H. Lopez-Vernaza M. Mellerowicz E. Perrot-Rechenmann C.. (2008). Dissecting the molecular basis of the regulation of wood formation by auxin in hybrid aspen. Plant Cell 20, 843–855. doi: 10.1105/tpc.107.055798 18424614 PMC2390731

[B53] Nishiyama R. Watanabe Y. Fujita Y. Le D. T. Kojima M. Werner T.. (2011). Analysis of cytokinin mutants and regulation of cytokinin metabolic genes reveals important regulatory roles of cytokinins in drought, salt and abscisic acid responses, and abscisic acid biosynthesis. Plant Cell 23, 2169–2183. doi: 10.1105/tpc.111.087395 21719693 PMC3160038

[B54] Oribe Y. Funada R. Shibagaki M. Kubo T. (2001). Cambial reactivation in locally heated stems of the evergreen conifer Abies sachalinensis (Schmidt) masters. Planta 212, 684–691. doi: 10.1007/S004250000430 11346941

[B55] Oribe Y. Kubo T. (1997). Effect of heat on cambial reactivation during winter dormancy in evergreen and deciduous conifers. Tree Physiol. 17, 81–87. doi: 10.1093/TREEPHYS/17.2.81 14759877

[B56] Ragni L. Nieminen K. Pacheco-Villalobos D. Sibout R. Schwechheimer C. Hardtke C. S. (2011). Mobile gibberellin directly stimulates Arabidopsis hypocotyl xylem expansion. Plant Cell 23, 1322–1336. doi: 10.1105/tpc.111.084020 21498678 PMC3101547

[B57] Ramachandran P. Wang G. Augstein F. De Vries J. Carlsbecker A. (2018). Continuous root xylem formation and vascular acclimation to water deficit involves endodermal ABA signalling *via* miR165. Dev. 145. doi: 10.1242/dev.159202 29361572

[B58] Rodriguez-Villalon A. Gujas B. Kang Y. H. Breda A. S. Cattaneo P. Depuydt S.. (2014). Molecular genetic framework for protophloem formation. Proc. Natl. Acad. Sci. U. S. A. 111, 11551–11556. doi: 10.1073/pnas.1407337111 25049386 PMC4128119

[B59] Rossi S. Deslauriers A. Anfodillo T. Carraro V. (2007). Evidence of threshold temperatures for xylogenesis in conifers at high altitudes. Oecologia 152, 1–12. doi: 10.1007/s00442-006-0625-7 17165095

[B60] Saito M. Kondo Y. Fukuda H. (2018). BES1 and BZR1 redundantly promote phloem and xylem differentiation. Plant Cell Physiol. 59, 590–600. doi: 10.1093/pcp/pcy012 29385529

[B61] Sehr E. M. Agusti J. Lehner R. Farmer E. E. Schwarz M. Greb T. (2010). Analysis of secondary growth in the Arabidopsis shoot reveals a positive role of jasmonate signalling in cambium formation. Plant J. 63, 811–822. doi: 10.1111/tpj.2010.63.issue-5 20579310 PMC2988407

[B62] Shi D. Lebovka I. López-Salmeroń V. Sanchez P. Greb T. (2019). Bifacial cambium stem cells generate xylem and phloem during radial plant growth. Development 146, dev171355. doi: 10.1242/dev.171355 30626594 PMC6340147

[B63] Shpak E. D. Berthiaume C. T. Hill E. J. Torii K. U. (2004). Synergistic interaction of three ERECTA-family receptor-like kinases controls Arabidopsis organ growth and flower development by promoting cell proliferation. Development 131, 1491–1501. doi: 10.1242/dev.01028 14985254

[B64] Smakowska-Luzan E. Mott G. A. Parys K. Stegmann M. Howton T. C. Layeghifard M.. (2018). An extracellular network of Arabidopsis leucine-rich repeat receptor kinases. Nat. 553, 342–346. doi: 10.1038/nature25184 PMC6485605 29320478

[B65] Smetana O. Mäkilä R. Lyu M. Amiryousefi A. Sánchez Rodríguez F. Wu M. F.. (2019). High levels of auxin signalling define the stem-cell organizer of the vascular cambium. Nature 565, 485–489. doi: 10.1038/s41586-018-0837-0 30626967

[B66] Smit M. E. McGregor S. R. Sun H. Gough C. Bågman A. M. Soyars C. L.. (2020). A PXY-mediated transcriptional network integrates signaling mechanisms to control vascular development in Arabidopsis. Plant Cell 32, 319–335. doi: 10.1105/tpc.19.00562 31806676 PMC7008486

[B67] Stewart J. J. Demmig-Adams B. Cohu C. M. Wenzl C. A. Muller O. Adams W. W. (2016). Growth temperature impact on leaf form and function in Arabidopsis thaliana ecotypes from northern and southern Europe. Plant Cell Environ. 39, 1549–1558. doi: 10.1111/pce.12720 26832121

[B68] Stewart W. N. Wilson N. Rothwell G. W. (1993) Paleobotany and the evolution of plants. (Cambridge University Press). Available online at: https://books.google.com/books/about/Paleobotany_and_the_Evolution_of_Plants.html?id=Fhm-oed74JgC (Accessed February 13, 2023).

[B69] Suer S. Agusti J. Sanchez P. Schwarz M. Greb T. (2011). WOX4 imparts auxin responsiveness to cambium cells in arabidopsis. Plant Cell 23, 3247. doi: 10.1105/tpc.111.087874 21926336 PMC3203433

[B70] Sundberg B. Uggla C. (1998). Origin and dynamics of indoleacetic acid under polar transport in Pinus sylvestris. Physiol. Plant 104, 22–29. doi: 10.1034/j.1399-3054.1998.1040104.x

[B71] Tonn N. Greb T. (2017). Radial plant growth. Curr. Biol. 27, R878–R882. doi: 10.1016/j.cub.2017.03.056 28898657

[B72] Truernit E. Bauby H. Dubreucq B. Grandjean O. Runions J. Barthélémy J.. (2008). High-resolution whole-mount imaging of three-dimensional tissue organization and gene expression enables the study of phloem development and structure in Arabidopsis. Plant Cell 20, 1494–1503. doi: 10.1105/tpc.107.056069 18523061 PMC2483377

[B73] Tuominen H. Puech L. Fink S. Sundberg B. (1997). A radial concentration gradient of indole-3-acetic acid is related to secondary xylem development in hybrid aspen. Plant Physiol. 115, 577–585. doi: 10.1104/pp.115.2.577 12223825 PMC158517

[B74] Turley E. K. Etchells J. P. (2022). Laying it on thick: a study in secondary growth. J. Exp. Bot. 73, 665–679. doi: 10.1093/jxb/erab455 34655214 PMC8793872

[B75] Uchida N. Lee J. S. Horst R. J. Lai H. H. Kajita R. Kakimoto T.. (2012). Regulation of inflorescence architecture by intertissue layer ligand-receptor communication between endodermis and phloem. Proc. Natl. Acad. Sci. U. S. A. 109, 6337–6342. doi: 10.1073/pnas.1117537109 22474391 PMC3341066

[B76] Uchida N. Tasaka M. (2013). Regulation of plant vascular stem cells by endodermisderived EPFL-family peptide hormones and phloemexpressed ERECTA-family receptor kinases. J. Exp. Bot. 64, 5335–5343. doi: 10.1093/jxb/ert196 23881395

[B77] Uggla C. Moritz T. Sandberg G. Sundberg B. (1996). Auxin as a positional signal in pattern formation in plants. Proc. Natl. Acad. Sci. U.S.A. 93, 9282–9286. doi: 10.1073/pnas.93.17.9282 11607701 PMC38633

[B78] Wang H. (2020). Regulation of vascular cambium activity. Plant Sci. 291. doi: 10.1016/j.plantsci.2019.110322 31928672

[B79] Wang N. Bagdassarian K. S. Doherty R. E. Kroon J. T. Connor K. A. Wang X. Y.. (2019). Organ-specific genetic interactions between paralogues of the PXY and ER receptor kinases enforce radial patterning in Arabidopsis vascular tissue. Development 146. doi: 10.1242/dev.177105 31043420

[B80] Wang J. Kucukoglu M. Zhang L. Chen P. Decker D. Nilsson O.. (2013). The Arabidopsis LRR-RLK, PXC1, is a regulator of secondary wall formation correlated with the TDIF-PXY/TDR-WOX4 signaling pathway. BMC Plant Biol. 13, 94. doi: 10.1186/1471-2229-13-94 23815750 PMC3716795

[B81] Wang Q. Little C. H. A. Odén P. C. (1995). Effect of laterally applied gibberellin A4/7 on cambial growth and the level of indole-3-acetic acid in Pinus sylvestris shoots. Physiol. Plant 95, 187–194. doi: 10.1111/j.1399-3054.1995.tb00826.x

[B82] Wang X. Mäkilä R. Mähönen A. P. (2023). From procambium patterning to cambium activation and maintenance in the Arabidopsis root. Curr. Opin. Plant Biol. 75, 102404. doi: 10.1016/j.pbi.2023.102404 37352651

[B83] Yang J. H. Lee K. H. Du Q. Yang S. Yuan B. Qi L.. (2020a). A membrane-associated NAC domain transcription factor XVP interacts with TDIF co-receptor and regulates vascular meristem activity. New Phytol. 226, 59–74. doi: 10.1111/nph.16289 31660587

[B84] Yang S. Wang S. Li S. Du Q. Qi L. Wang W.. (2020b). Activation of ACS7 in Arabidopsis affects vascular development and demonstrates a link between ethylene synthesis and cambial activity. J. Exp. Bot. 71, 7160–7170. doi: 10.1093/jxb/eraa423 32926140

[B85] Ye L. Wang X. Lyu M. Siligato R. Eswaran G. Vainio L.. (2021). Cytokinins initiate secondary growth in the Arabidopsis root through a set of LBD genes. Curr. Biol. 31, 3365–3373.e7. doi: 10.1016/j.cub.2021.05.036 34129827 PMC8360765

[B86] Zhang J. Eswaran G. Alonso-Serra J. Kucukoglu M. Xiang J. Yang W.. (2019). Transcriptional regulatory framework for vascular cambium development in Arabidopsis roots. Nat. Plants 5, 1033–1042. doi: 10.1038/s41477-019-0522-9 31595065 PMC6795544

[B87] Zhang H. Lin X. Han Z. Qu L.-J. Chai J. (2016a). Crystal structure of PXY-TDIF complex reveals a conserved recognition mechanism among CLE peptide-receptor pairs. Cell Res. 26, 543–555. doi: 10.1038/cr.2016.45 27055373 PMC4856767

[B88] Zhang H. Lin X. Han Z. Wang J. Qu L. J. Chai J. (2016b). SERK family receptor-like kinases function as co-receptors with PXY for plant vascular development. Mol. Plant 9, 1406–1414. doi: 10.1016/j.molp.2016.07.004 27449136

